# *Rif1* Regulates Self-Renewal and Impedes Mesendodermal Differentiation of Mouse Embryonic Stem Cells

**DOI:** 10.1007/s12015-023-10525-1

**Published:** 2023-03-27

**Authors:** Cheng Liu, Peng Yu, Zongna Ren, Fang Yao, Li Wang, Guang Hu, Pishun Li, Quanyi Zhao

**Affiliations:** 1 State Key Laboratory of Cardiovascular Disease, Key Laboratory of Application of Pluripotent Stem Cells in Heart Regeneration, Fuwai Hospital, National Center for Cardiovascular Diseases, Chinese Academy of Medical Sciences and Peking Union Medical College, 167 North Lishi Road, 100037 Beijing, China; 2 Shenzhen Key Laboratory of Cardiovascular Disease, Fuwai Hospital Chinese Academy of Medical Sciences, 518057 Shenzhen, China; 3 College of Veterinary Medicine, Hunan Agricultural University, 410128 Changsha, China; 4 Epigenetics and Stem Cell Biology Laboratory, National Institute of Environmental Health Sciences, 2779 Research Triangle Park, NC USA

**Keywords:** *Rif1*, Mouse embryonic stem cells, Germ layer differentiation, Histone modifications, ERK signaling pathway

## Abstract

**Background:**

RAP1 interacting factor 1 (*Rif1*) is highly expressed in mice embryos and mouse embryonic stem cells (mESCs). It plays critical roles in telomere length homeostasis, DNA damage, DNA replication timing and ERV silencing. However, whether *Rif1* regulates early differentiation of mESC is still unclear.

**Methods:**

In this study, we generated a *Rif1* conditional knockout mouse embryonic stem (ES) cell line based on Cre-loxP system. Western blot, flow cytometry, quantitative real-time polymerase chain reaction (qRT-PCR), RNA high-throughput sequencing (RNA-Seq), chromatin immunoprecipitation followed high-throughput sequencing (ChIP-Seq), chromatin immunoprecipitation quantitative PCR (ChIP-qPCR), immunofluorescence, and immunoprecipitation were employed for phenotype and molecular mechanism assessment.

**Results:**

*Rif1* plays important roles in self-renewal and pluripotency of mESCs and loss of *Rif1* promotes mESC differentiation toward the mesendodermal germ layers. We further show that *Rif1* interacts with histone H3K27 methyltransferase EZH2, a subunit of PRC2, and regulates the expression of developmental genes by directly binding to their promoters. *Rif1* deficiency reduces the occupancy of EZH2 and H3K27me3 on mesendodermal gene promoters and activates ERK1/2 activities.

**Conclusion:**

*Rif1* is a key factor in regulating the pluripotency, self-renewal, and lineage specification of mESCs. Our research provides new insights into the key roles of *Rif1* in connecting epigenetic regulations and signaling pathways for cell fate determination and lineage specification of mESCs.

**Graphical abstract:**

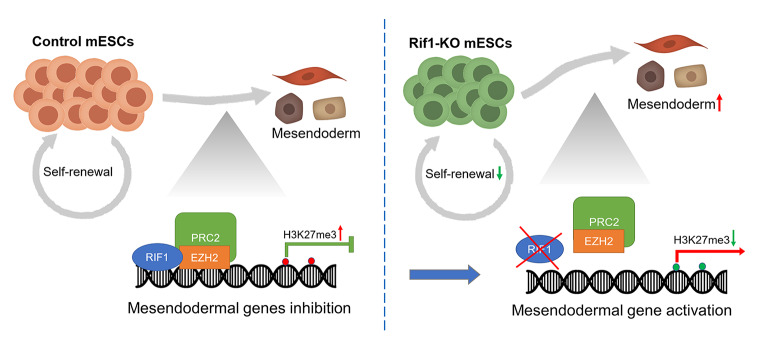

**Supplementary Information:**

The online version contains supplementary material available at 10.1007/s12015-023-10525-1.

## Introduction

Embryonic stem cells (ESCs) derive from the inner cell mass of blastocyst, an early-stage pre-implantation embryo. Self-renewal and pluripotency are their two defining features. Stem cell identity and differentiation are largely regulated by histone modifications and chromatin remodeling. Polycomb group (PcG) is a set of chromatin modifiers that play a central role in maintaining cellular identity and silencing developmental genes [1].

Mammalian PcG proteins are classified into two main categories, polycomb repressive complex 1 (PRC1) and 2 (PRC2), depending on their biological and functional features. Specifically, PRC2 occupies the promoters of developmental genes in ESCs [2]. PRC2 is composed of four core proteins, SUZ12, EED, RBBP4/7, and either of the two histone H3K27 methyltransferases, EZH1 or EZH2, which catalyzes mono-, di-, and tri-methylation of histone H3 on lysine 27 (H3K27me1/2/3) [3, 4]. More specifically, EZH2 suppresses developmental gene expression by catalyzing histone H3 methylation on lysine 27(H3K27me3) [5]. Therefore, loss of EZH2 leads to embryonic lethality due to gastrulation defects [6]. EZH2 deficiency also leads to defects in self-renewal and differentiation in mouse ESCs (mESCs) [7], while exclusively expressing EZH2 variant, ex14D-EZH2, in ES cells promotes the expression of mesoderm genes [8].

The early stages of embryonic development are regulated by the activities of extracellular signal-regulated kinase (ERK)/mitogen-activated protein kinase (MAPK) signal-transduction cascade, which mediate the effects of growth factors by sequentially activating the Ras-like GTPase, Raf kinase, serine/threonine protein kinase MEK, and ERK. ERK/MAPK signaling pathways are key regulatory mechanisms of cell-cycle progression, proliferation, differentiation, and carcinogenesis [9, 10]. ERK signaling inactivation constrains the differentiation of mESCs [11, 12]. Meanwhile, activation of MEK/ERK signaling promotes the differentiation of human embryonic stem cells (hESCs) [13]. It was recently reported that inhibiting MAPK/ERK kinase (MEK) and glycogen synthase kinase 3 (GSK3) could maintain pluripotency and proliferation abilities of mESCs, which was called 2i medium method [14]. Also, it has been revealed that ERK1/2 plays a direct role in modulating chromatin features required for regulating developmental gene expression. ERK2 regulates mESC differentiation by cooperating with PRC2 and phosphorylation of RNA polymerase II at Ser5 [15].

RAP1 interacting factor 1 (*Rif1*) was originally identified as a telomere-associated protein in budding yeast [16]. It regulates the yeast telomere length homeostasis by regulating telomerase recruitment to telomeres [17, 18]. *Rif1* has also been shown to play roles in the DNA damage response [19–25], DNA replication timing [26–28], and epigenetic gene regulation [29, 30] and is required for ESC maintenance as a factor involved in pluripotency network [31–34]. Deletion of *Rif1* leads to early embryonic lethality in C57BL/6J mice [35], suggesting that *Rif1* is critical for early embryo development, while whether *Rif1* regulates the early differentiation of mESCs remains elusive.

Our studies investigated the role of *Rif1* in the self-renewal and early differentiation of mESCs. We created *Rif1*-knockout mESCs (*Rif1*-KO mESCs) via CRISPR Cas9 gene editing. Our results suggest that *Rif1* knockout leads to the initiation of differentiation toward mesendodermal lineage. Furthermore, RIF1 interacts with EZH2, a subunit of PRC2 and represses the transcription. *Rif1* deficiency in mESCs impairs proliferation and pluripotency and induces mesendodermal differentiation by activating the ERK1/2 signaling and reducing the occupancy of EZH2 and H3K27me3 on mesendodermal gene promoters.

## Materials and Methods

### Cell Culture

E14 mESCs were obtained from the American Type Culture Collection. Rosa26-CreERT2 ESCs were kindly provided by Shaun Cowley. Inducible Rif1 knockout and HA-tagged Rif1 ESCs were maintained in our laboratory. mESCs were cultured on 0.1% gelatin coated plates in the 2i medium (for experiments) as described before [14]. 2i medium contains N2B27 medium supplemented with 2i (PD0325901, 1 µM and CHIR99021, 3 µM, selleck) + LIF (1000 U/ml, Millipore).

### EB Formation

mESCs were trypsinized, re-suspended in EB medium (DMEM supplemented with 10% FBS (Gibco ES Cell FBS Qualified), 0.1 mM non-essential amino acids, 2 mM Glutamine, and 100 U/ml penicillin/streptomycin) at a cell density of 1.0-1.5 × 10^5^ cells/ml and plated on an Ultra-Low Attachment Multiple Well Plate (Corning, 3473).

### RNA Isolation, Reverse Transcription, and Real-Time PCR

Total RNA was isolated from cells using the GeneJet RNA purification kit (Thermo Scientific), and 0.5 mg total RNA was reverse transcribed to generate cDNA using the iScript cDNA Synthesis Kit (Bio-Rad) according to manufacturer’s instructions. qPCRs were performed using iTaq Universal SYBR Green Supermix (Bio-Rad) on the QuantStudio 6 and 7 Flex Real-Time PCR Systems. *Gapdh* was used for normalization. Primers used in the study are listed in additional file 1: Table S1. For RNA-seq, libraries were prepared from two biological replicates using the KAPA mRNA HyperPrep Kit and sequenced on the NextSeq (Illumina).

### Teratoma Formation Assay

2 × 10^7^ Cre-ERT2 and Rif1-KO ES cells were trypsinized, washed with PBS and suspended in total 500ul PBS, then injected subcutaneously into the 8-week-old SCID/beige mice. Each mouse was injected at 2 spots (2 × 10^6^ mESCs/spot), Cre-ERT2 mESCs on one side and Rif1-KO mESCs on the other side. Four weeks after injection, the SCID mice were killed and tumors were harvested. Teratomas were homogenized for total RNA extraction.

For preparation of teratoma paraffin blocks, teratomas were transferred into 4% PFA overnight at 4 °C. 5-µm-thick paraffin sections were deparaffinized and stained with hematoxylin and eosin (H&E). All paraffin-sections were subjected to histological examination after H&E staining.

### Western Blot and Immunofluorescence Staining

Cells were lysed with RIPA. Cell lysate was loaded into a NuPAGE® Bis-Tris gels (4–12%) or NuPAGE® Tris-Acetate gels (3–8%, for > 250 KD large proteins) and transferred onto a PVDF membrane. The membrane was blocked with 5% non-fat milk at room temperature for 2 h, followed by incubation with primary antibodies at 4℃ overnight. The blot was subsequently incubated with either horse-radish peroxidase (HRP)-conjugated anti-mouse IgG or HRP-conjugated antirabbit IgG. Signals were detected using Amersham ImageQuant 800.

Cells were fixed using 4% paraformaldehyde (PFA) at room temperature for 15 min, followed by 5% bovine serum albumin blocking with 0.3% Triton X-100 for 30 min. They were then incubated with primary antibodies at 4℃ overnight, followed by secondary antibodies (Life Technologies). Nuclei were counter stained with DAPI (Sigma). Confocal images were taken on the LEICA SP8 LIGHTNING microscope. All experiments were performed three or more times, and representative results were shown in the figures.

### ChIP-qPCR and ChIP-Seq Sample Preparation

Rif1 ChIP was performed as described previously [30]. Cre-ERT2 and Rif1-KO cells were fixed using 1% formaldehyde for 10 min and then treated with 0.125 M glycine for 5 min to stop the fixation. Then the cells were harvested, and DNA was fragmented to 300–500 bp by sonication with Covaris S220 sonicator. Immunoprecipitation was performed with 3 mg Dynabeads protein G (Life Technologies) conjugated rabbit monoclonal anti-Rif1 antibody overnight at 4℃. Afterward, beads were washed, eluted, and reverse cross-linked. DNA was extracted by phenol/chloroform and precipitated. The resulting DNA was analyzed with qPCR using indicated primers and data were presented as the percentage of input (Additional file 1: Table S1).

### Immunoprecipitation (IP)

E14 or HA-Rif1 mESCs were used for immunoprecipitation (IP). Cells were harvested and lysed with lysis buffer (150 mM NaCl, 50 mM Tris, 1% NP40, pH 8.0, 10mM NaF, 1mM Na_3_VO_4_, Roche ethylenediaminetetraacetic acid (EDTA)-free protease inhibitor, phenylmethylsulfonyl fluoride (PMSF)). IP was carried out using anti-HA (3F10, Roche 11,815,016,001) beads or beads conjugated with anti-Rif1 antibody overnight at 4℃. After IP, beads were washed with lysis buffer and bound proteins were eluted with 2*loading buffer (no DTT) at 50℃ for 10 min. DTT was then added to a final concentration of 100 mM and incubated for another 5 min at 95℃. All experiments were performed three or more times, and representative results were shown in the figures.

### Bioinformatics Analysis

#### RNA-seq Analysis

The reads were aligned to genome (mm9) with STAR (v020201) and annotated to Ref Seq genes using feature Counts (v1.6.2). Genes with reads less than 50 in all samples were removed. The DEG analysis was conducted by edge R (v3.28.1) with cutoff of p-value 0.05 and FC > 1.5. Enrichment analysis was done by GSEA 3.0 and cluster Profiler (v3.14.3).

#### ChIP-seq Analysis

ChIP-seq reads were aligned to mm9 genome and the peaks were called with SICER2 (v1.0.2, -w 200 -g 600 --false_discovery_rate 0.01). Differential peaks were detected by sicer_df (fdr < 1e-6 and fold change > 1.5). For each gene, the peak with lowest FDR q value was selected as the representative peak. BigWig files were created by deepTools (v3.2.0) and viewed in IGV (v2.3.92). The Rif1 binding motif was discovered by MEME-ChIP algorithm and the de novo co-binding motifs were scanned by HOMMERfindMotif Genome.pl module with default configurations.

### Antibodies

Antibodies used in this study include: HA (C29F4, CST 3724), RIF1 (Santa Cruz, SC-65,191), EZH2 (CST, 5246), SETDB1 (Santa Cruz, sc-66,884), H3K9me3 (Abcam, ab8898), H3K4me3 (Active motif, 39,159), H3K27me3 (Active motif, 39,155), H3K9ac (Millipore, 07-352), H3K27ac (Active motif, 31,933), Suv39H1 (CST, 8729), EHMT2 (CST, 3306).

### Data Analysis and Statistics

Statistical analyses were carried out using GraphPad Prism 9.0 software. All experiments were carried out in at least three independent biological replicates for each group. All data are presented as mean ± standard error of the mean (SEM). Statistical significance between two groups was evaluated with unpaired Student’s t-test. For three or more groups, statistical analyses were performed using one-way ANOVA followed by Bonferroni post-hoc analysis. *p < 0.05, **p < 0.01, ***p < 0.001. *p* < 0.05 was considered statistically significant.

## Results

### *Rif1* is Essential for Maintaining Stem Cell Identity in mESCs

**Fig. 1:**
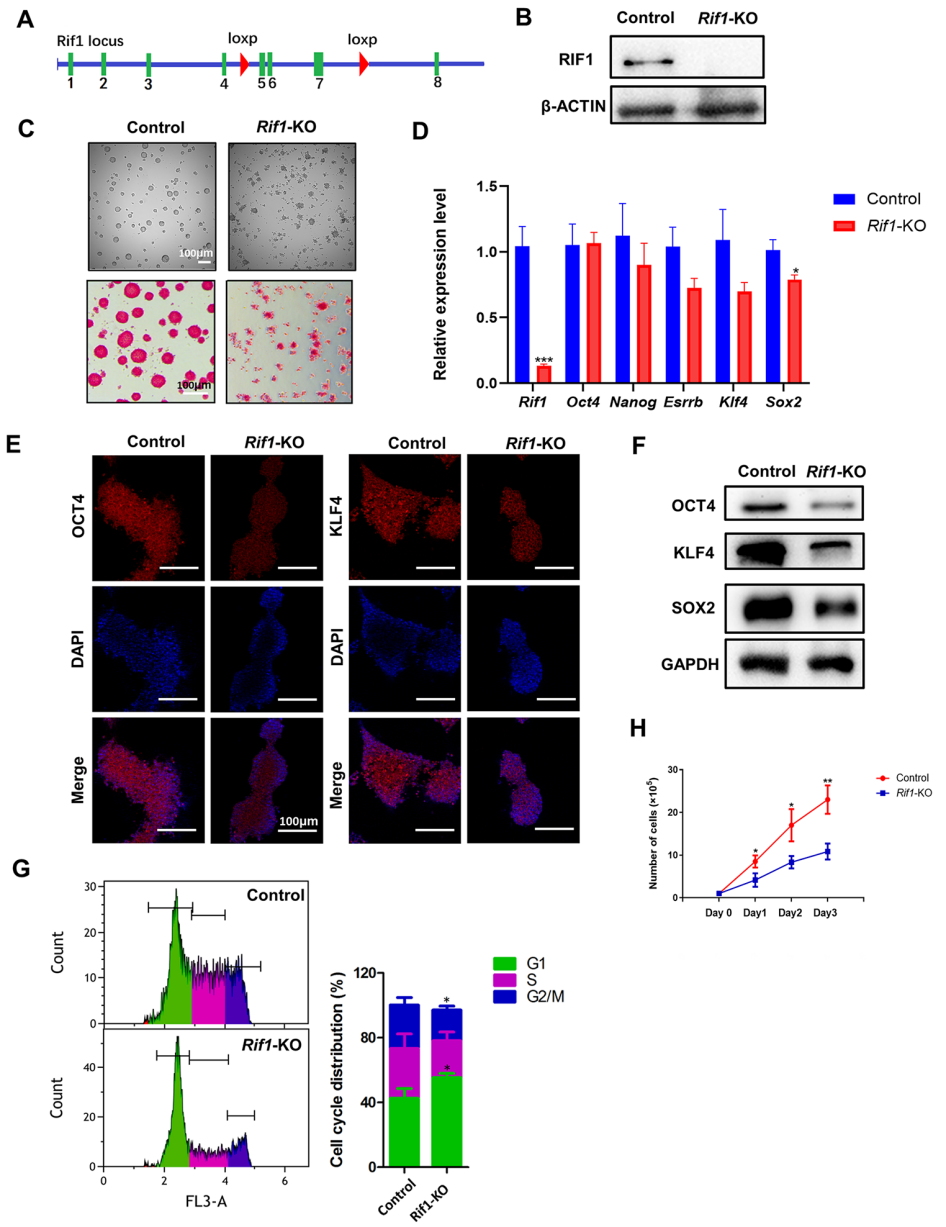
***Rif1 *is required for maintaining mESC identity**. **(A)** Schematic illustration of the conditional deletion of *Rif1* in mESCs. **(B)** Immunoblot analysis of RIF1 protein level in control and *Rif1*-KO mESCs that both had been treated with 4-OHT for 48 h. **(C)** Alkaline phosphatase (AP) staining and phase contrast images of mESCs colonies. Scale bars, 100 μm. **(D)** The relative mRNA levels of pluripotency markers in control and *Rif1*-KO mESCs were measured via qRT-PCR (n = 5). *p < 0.05 compared with control; t test. **(E)** Control and *Rif1*-KO mESCs were immunofluorescently stained for Oct4 and Klf4 expression and nuclei were counterstained with DAPI. Scale bars, 100 μm. **(F)** The protein levels of OCT4, KLF4, and SOX2 in control and *Rif1*-KO mESCs. **(G)** Cell-cycle analysis of control and *Rif1*-KO mESCs. Representative histograms show distribution of cells in sequential phases (G0/G1, green; S, purple; and G2/M, blue) of cell cycle. Bar chart shows the percentage of cell population (n = 3, 20,000 cells per sample). *p < 0.05 compared with control; t test. **(H)** Control and *Rif1*-KO mESCs were seeded into gelatin-coated wells (1 × 10^5^ cells per well) and proliferation was evaluated by monitoring cell counts over the ensuing 3-day culture period (n = 3). *p < 0.05; **p < 0.01 compared with control; t test. Data were collected from three independent replicates and are shown as means ± SEM.

In previous studies, we generated a *Rif1* conditional knockout mouse embryonic stem (ES) cell line [30], which has three coding exons of the *Rif1* gene deleted by Cyclization Recombination Enzyme (Cre), based on Cre-loxP system (Fig. 1A). *Rif1* was completely removed after the cells were treated with 4-OHT (0.1 µM) for 2 days and cultured for another day without treatment. And we confirmed the depletion of RIF1 proteins before collecting the cells for further analysis (Fig. 1B). To exclude the effects of Cre-mediated toxicity, we used Cre-ERT2 cell line as a negative control, which has no loxp flanking sites on the exons of *Rif1*. Compared to the control, loss of *Rif1* reduced colony size and resulted in incomplete colony edge. The alkaline phosphatase (AP) activity lowered significantly in *Rif1*-KO mESCs (Fig. 1C). The mRNA levels of the pluripotency factors remained unchanged except for Sox2 (Fig. 1D). However, *Rif1*-KO mESCs showed reduced protein levels of the pluripotency factors, such as SOX2, OCT4, and KLF4 (Fig. 1E, F). It was reported that RIF1 has protein-protein interactions with the pluripotency factors [36, 37], so we investigated whether *Rif1* knockout induces the protein degradation of these factors. We performed Western Blot to evaluate the protein level of OCT4 in the proteasome inhibitor MG132 treated *Rif1*-KO mESCs and found that the reduction of the protein level of OCT4 induced by *Rif1*-KO could be restored with MG132 treatment (additional file 1: Fig. S1A). In addition, *Rif1*-KO mESCs showed reduced proliferation rate (Fig. 1H and additional file 1: Fig. S1B) and the cell cycle analysis indicated that *Rif1*-KO mESCs had a higher proportion of G1 cells than Cre-ERT cells did (Fig. 1G). Consistent with it, we found decreased expression of CDK2 and increased expression of P21, a potent inducer of G1 arrest (Additional file 1: Fig. S1C).

Collectively, these results suggest that *Rif1* contributes to the maintenance of stem cell identity and proliferation.

### *Rif1* Deficiency Results in Aberrant Expression of differentiation-associated Genes

**Fig. 2:**
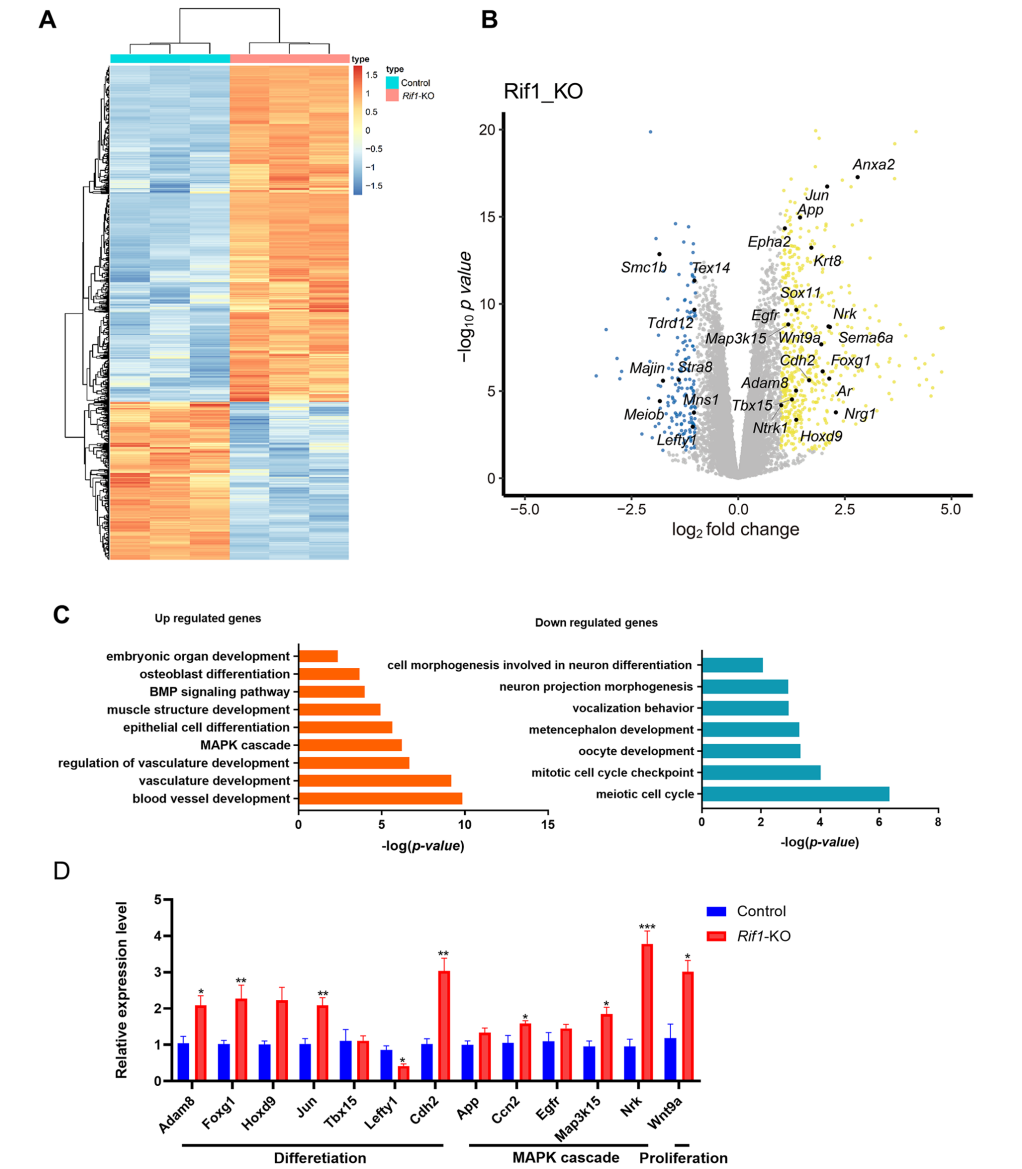
**Loss of *Rif1* results in aberrant expression of differentiation-associated genes**. **(A)** Heatmap illustrating the RNA expression level of differentially expressed genes in control and *Rif1*-KO mESCs (FC > 1.5 and p < 0.05). **(B)** Volcano plot showing the distribution of the differentially expressed genes with 1-fold changes upon *Rif1* deletion. **(C)** Gene ontology (GO) enrichment analysis for genes differentially expressed upon *Rif1* deletion. Analysis was carried out using Metascape. **(D)** Validation of RNA-seq data by qRT-PCR analysis. The relative mRNA levels of indicated differentiation-related genes, MAPK cascade-related genes and *Wnt9a* (proliferation-related gene) in control and *Rif1*-KO mESCs (n = 3). *p < 0.05; **p < 0.01;***p < 0.001 compared with control;t test. Data were collected from three independent replicates and are shown as means ± SEM.

To explore the molecular basis of phenotypic alterations observed in *Rif1*-KO mESCs, we profiled the global gene expression in Cre-ERT2 and *Rif1*-KO mESCs by RNA-sequencing (RNA-seq). Our data showed that loss of *Rif1* increased the expression of 1179 genes and reduced the expression of 655 genes (fold change > 1.5, p < 0.05; Fig. 2A, B and additional file 2: Table S2), which is consistent with previous studies showing that *Rif1* is a transcription repressor [29]. Gene Ontology (GO) analysis of down-regulated gene sets indicated that these genes were associated with cell cycle, including *Smc1b*, *Majin*, *Lefty1*. Among up-regulated genes, our analysis revealed that a set of genes were involved in differentiation and MAPK signaling pathway, including *Wnt9a*, *Ma3pk15*, *Sema6a*, *Jun*, *Hoxd9*, *Tbx15* (Fig. 2B), as well as MAPK and BMP signaling pathways which are also involved in development and differentiation (Fig. 2C). These genes observed by RNA-seq analysis were significantly reduced or increased in *Rif1*-KO mESCs (Fig. 2D).

Altogether, the evidence collected clearly suggests that *Rif1* has key roles in controlling the cell cycle and differentiation of mESCs.

### *Rif1*-KO Promotes Mesendodermal Gene Expression in mESCs

**Fig. 3:**
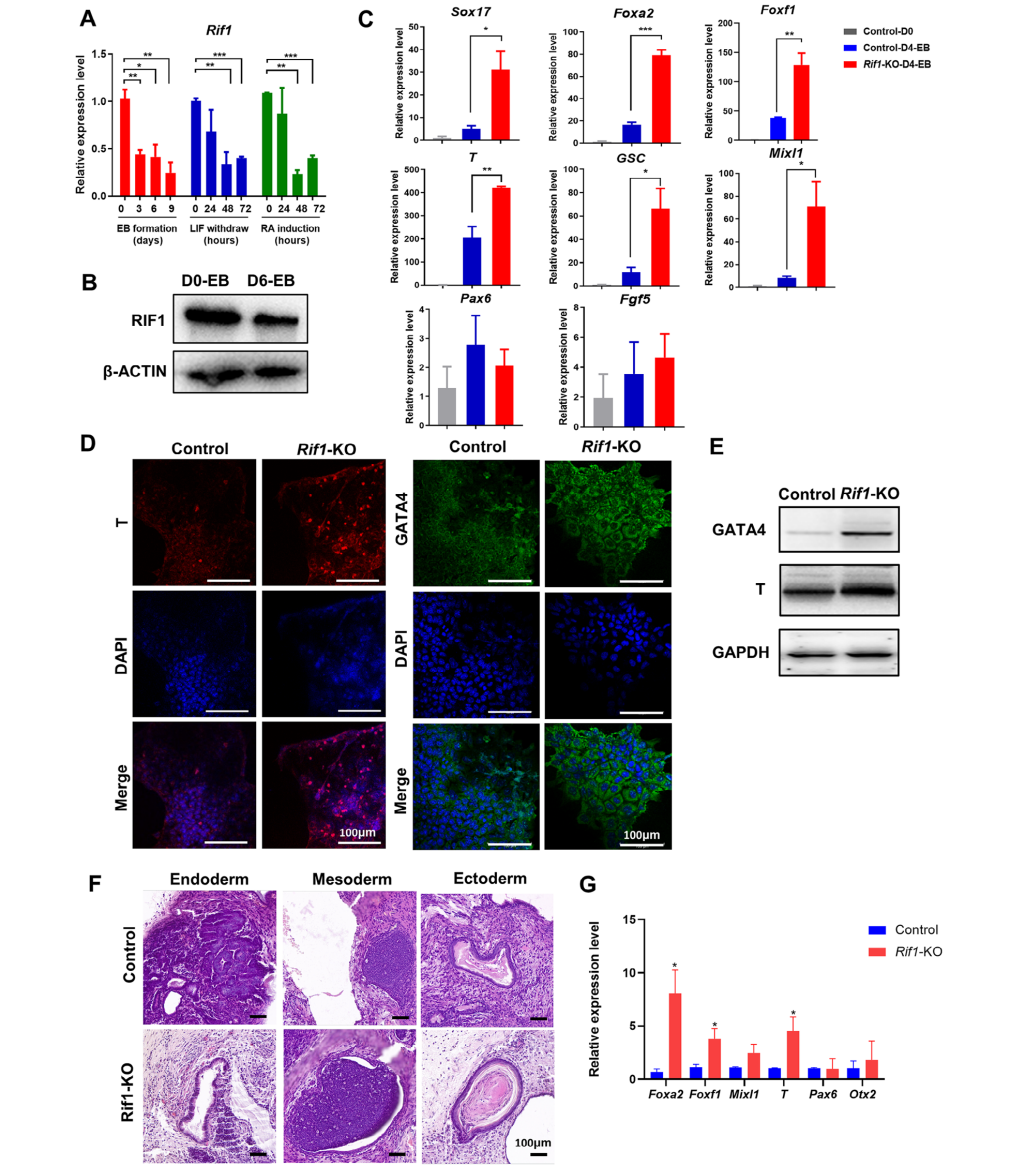
***Rif1*-KO promotes mesendodermal gene expression in mESCs. (A)** The relative mRNA expression of *Rif1* during ESC differentiation induced by LIF-withdrawal, retinoic acid (RA) treatment and embryoid body (EB) formation via qRT-PCR. **(B)** RIF1 protein levels were evaluated in control and *Rif1*-KO EBs via Western blot. **(C)** The relative mRNA expression of ectoderm, mesoderm, and endoderm markers in control and *Rif1*-KO mESCs via qRT-PCR at Day 4 after embryonic body (EB) differentiation. Expression is normalized by *Gapdh* (n = 3). *p < 0.05; **p < 0.01 compared with control mESCs at Day 4; one-way analysis of variance. **(D)** Control and *Rif1*-KO mESCs were immunofluorescently stained for GATA4 (green) and T (red) expression, and nuclei were counterstained with DAPI (blue). Scale bars, 100 μm. **(E)** GATA4 and T protein levels were evaluated in control and *Rif1*-KO mESCs via Western blot. **(F)** The morphology and H&E staining of teratoma derived from control and *Rif1*-KO mESCs. All three germ layer tissues were present on the teratoma. Scale bars, 100 μm. (G) mRNA levels of lineage marker genes in teratomas from mice that had been injected with control, or *Rif1*-KO mESCs were measured via qRT-PCR (n = 3). *p < 0.05 compared with control; t test. Data were collected from three independent replicates and are shown as means ± SEM.

Previous publications indicate that *Rif1* is related to differentiation of mESCs toward the neural lineage [29]. Our RNA-seq results also showed that *Rif1* is required for differentiation. However, whether *Rif1* regulates the early differentiation of mESCs is unknown. In agreement with its role in self-renewal, the mRNA and protein levels of *Rif1* were downregulated during mESCs differentiation (Fig. 3A, B). To investigate the effects of loss of *Rif1* on mESCs germ layer differentiation, embryoid bodies (EBs) were used as in vitro differentiation models. The sizes of *Rif1* knockout embryoid bodies were significantly smaller than those of controls (Additional file 1: Fig. S2A). Next, we evaluated the expression of lineage markers on day 4 of mESC differentiation. In *Rif1*-KO mESCs, mRNA levels of mesoderm markers (*T*, *GSC*, *Foxf1* and *Mixl1*) and endoderm markers (*Foxa2* and *Sox17*) gradually increased after 4 days of spontaneous EB differentiation, whereas neuroectoderm markers expressions did not change in *Rif1*-KO mESCs compared with those in Cre-ERT2 mESCs (Fig. 3C). GATA4 and T also exhibited much higher expression levels in *Rif1*-KO mESCs evaluated by both immunofluorescence (Fig. 3D) and Western blot (Fig. 3E).

Although both WT and *Rif1*-KO mESCs could form teratomas that contain all three germ layers (endoderm, mesoderm, and ectoderm) when injected into adult severe combined immunodeficient (SCID) mice (Fig. 3F), the sizes of teratomas formed by *Rif1*-KO mESCs were smaller than those by WT cells (Additional file 1: Fig. S2B). However, *Rif1*-KO promoted the development of three germ layers formation in teratomas. First, we quantified the area of each germ layer in all images, showing that the mesendodermal area was significantly increased in *Rif1*-KO teratomas (Additional file 1: Fig. S2C). Further, to check the molecular signature of mesendodermal layer, we evaluated the mRNA levels of the mesendodermal markers such as *Foxa2, T*, and *Foxf1*, showing that the expression of these genes was upregulated in the *Rif1*-KO teratomas, while the expression of ectodermal makers *Pax6* and *Otx2* had no significant changes (Fig. 3G). We also confirmed that the protein level of the representative maker FOXA2, was elevated in *Rif1*-KO samples (Additional file 1: Fig. S2D).

Together, these data indicate that *Rif1* knockout promotes mesendodermal gene expression and germ layer development in mESCs.

### RIF1 Regulates differentiation-associated Genes Through Directly Binding to their Promoters

**Fig. 4:**
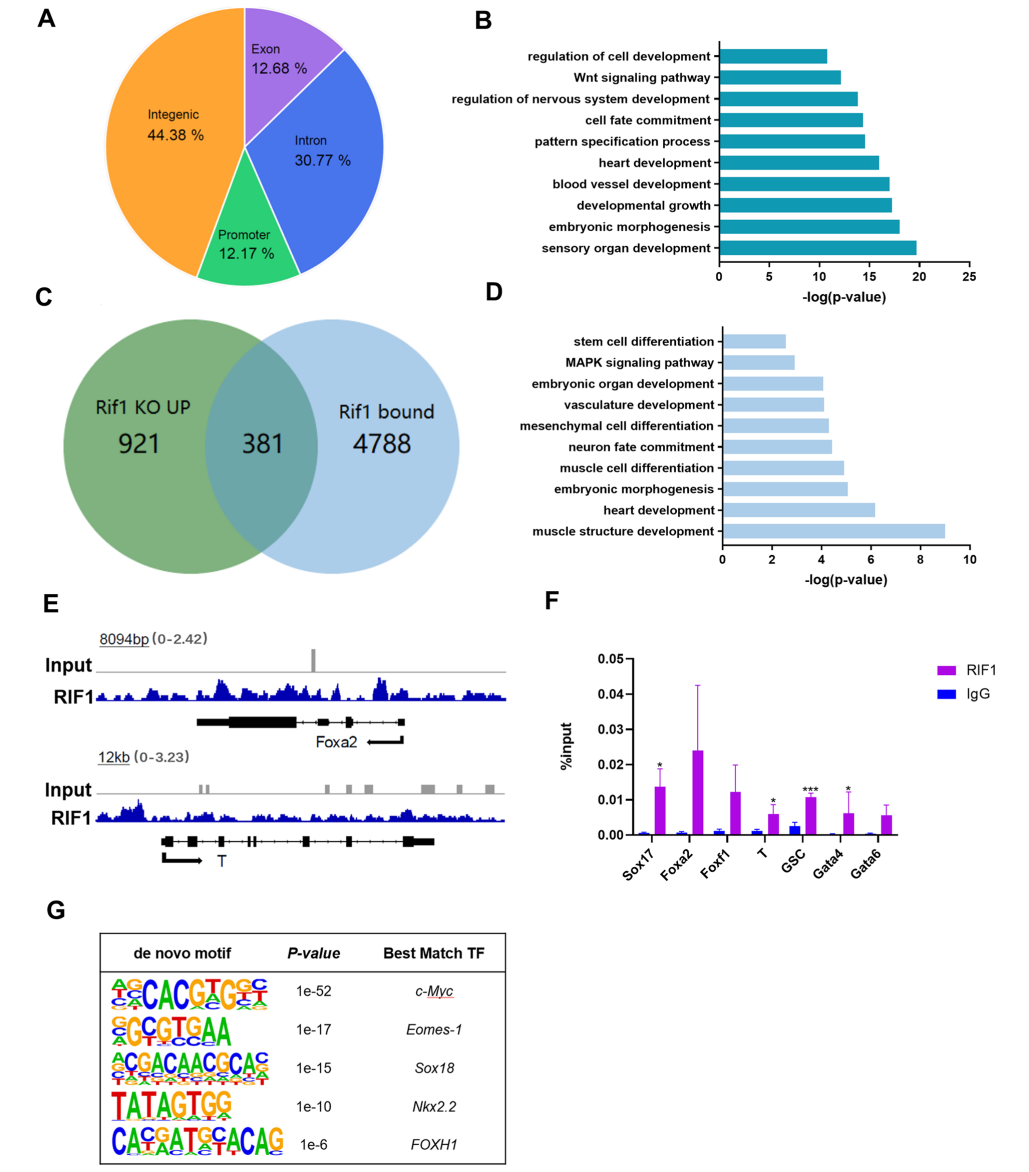
**RIF1 regulates differentiation-related genes through directly binding to their promoters**. **(A)** Pie chart showing the genomic distribution of RIF1 binding sites in mESCs. **(B)** Gene ontology (GO) enrichment analyses fo r genes that RIF1 binds to. **(C)** Venn diagram showing the overlap between genes up-regulated by RIF1 deletion with FC > 1.5 and genes occupied by RIF1. **(D)** GO analysis for biological processes associated with the overlapping genes. **(E)** Genome tracks showing RIF1 occupancy near lineage-specific markers for endoderm and mesoderm. **(F)** Verification of the ChIP-Seq results by ChIP-qPCR (n = 3). *p < 0.05; ***p < 0.001 compared with IgG; t test. **(G)** De novo motif analysis of occupancy peaks by RIF1. Data were collected from three independent replicates and are shown as means ± SEM.

In order to further explore the mechanism of differentiation regulated by *Rif1*, we analyzed published RIF1 ChIP sequencing (ChIP-seq) data in mESCs [30], which identified that RIF1 bound both intergenic and intragenic regions (Fig. 4A). Genes which have RIF1 bound to their promoter regions had a strong association with embryonic morphogenesis, cell fate commitment, and regulation of cell differentiation (Fig. 4B). Almost 40% of the differentially up-regulated genes in RNA-seq also had direct RIF1 binding, indicating that these genes are potential RIF1 target genes (Fig. 4C). GO analysis of these overlapping genes showed significant enrichment in embryonic morphogenesis and stem cell differentiation-related terms (Fig. 4D). We analyzed the RIF1 binding regions located on or near these 381 genes and found that RIF1 was more likely to bind their promoter regions, though when taking all RIF1 binding peaks into analysis, this tendency was not observed (Fig. 4A and additional file 1: Fig. S2E). Then we focused on the genes with RIF1 bound to their promoter regions, we identified 789 genes and 96 out of these showed up-regulated mRNA level in RNA-seq data (Additional file 1: Fig. S2F). We further annotated the function of these overlapping genes and found that they had significant enrichments on cell cycle, development and stem cell differentiation, which was consistent with all RIF1 targets (Additional file 1: Fig. S2G). The genome sessions of the individual genes and ChIP followed by quantitative PCR (ChIP-qPCR) also confirmed that RIF1 occupied genomic regions of the mesendodermal genes (Fig. 4E, F). Notably, we obtained an AC-rich 11-bits motif with E value 8.9E-84 of the RIF1 binding regions as its potential binding motif (Additional file 1: Fig. S2H). Furthermore, the de novo motif analysis showed that the binding motifs of *c-Myc*, *Emos-1*, *Sox18*, *FoxH1* and *Nkx 2.2*, which are key regulators of mESCs division and differentiation, were significantly enriched with RIF1 (Fig. 4G).

Altogether, the transcription of differentiation-associated genes in mESCs is regulated by RIF1 through direct promoter binding.

### RIF1 Suppresses Mesendodermal gene Expression in mESCs by Promoting EZH2-Catalyzed Trimethylation of H3K27

**Fig. 5:**
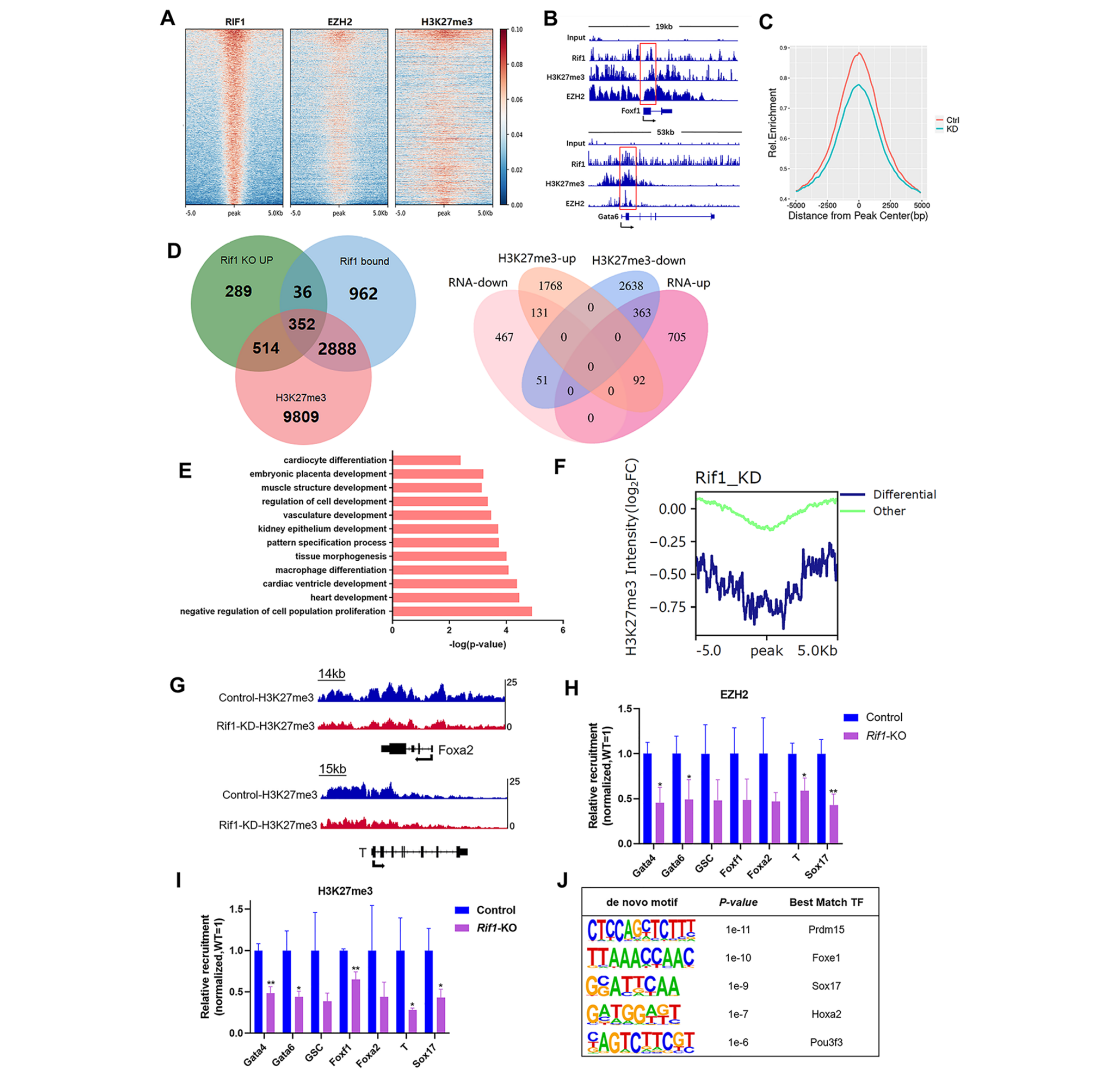
**RIF1 suppresses mesendodermal gene expression via EZH2-catalyzed trimethylation of H3K27 in mesendodermal gene promoters**. **(A)** The heatmap of RIF1, EZH2, and H3K27me3 at genomic regions surrounding (± 5 kb) RIF1 peak center in mESCs from published ChIP-seq data. **(B)** Genome tracks showing RIF1, EZH2 and H3K27me3 occupancy near lineage-specific markers for endoderm (*Gata6*) and mesoderm (*Foxf1*). **(C)** Comparation of H3K27me3 ChIP-seq intensity surrounding (± 5 kb) H3K27me3 peak center after *Rif1* knockdown. **(D)** Venn diagram showing overlap between genes upregulated by Rif1 loss and Rif1-occupied, H3K27me3-occupied genes (left panel). Venn diagram showing the overlap between genes differentially regulated by *Rif1* deletion with FC > 1.5 and genes that have decreased occupancy of H3K27me3(right panel). **(E)** GO analysis for biological processes associated with the genes that are both up-regulated in RNA-seq and less occupied by H3K27me3. **(F)** Comparation of RPM normalized H3K27me3 ChIP-seq intensities on H3K27me3 peaks center with ± 5 kb extension between differential genes and other genes after *Rif1* knockdown. **(G)** Genome tracks showing differential occupancy by H3K27me3 near lineage-specific markers for endoderm and mesoderm. **(H)** The binding of promoter sequences for mesendodermal genes to EZH2 was evaluated in control and *Rif1*-KO mESCs via ChIP-qPCR; results were normalized to control cells (n = 3). *p < 0.05; **p < 0.01 compared with control; t test. **(I)** The binding of promoter sequences for mesendodermal genes to H3K27me3 was evaluated in control and *Rif1*-KO mESCs via ChIP-qPCR; results were normalized to control cells (n = 3). *p < 0.05; **p < 0.01 compared with control; t test. **(J)** De novo motif analysis of decreased occupancy peaks by H3K27me3. Data were collected from three independent replicates and are shown as means ± SEM.

Previous study indicates that RIF1 is an epigenetic modifier that interacts with EZH2, a H3K27 methyltransferase [30]. To understand how the interactions and H3K27me3 contribute to the transcriptional regulations of genes involved in mESC differentiation, we further analyzed the published ChIP-seq data of RIF1, EZH2 and H3K27me3. Heatmap clustering of the three datasets on RIF1 peaks indicated that RIF1 and EZH2 colocalized broadly across the genome (Fig. 5A and additional file 1: Fig. S3A) and co-occupied genomic regions near mesendodermal genes (Fig. 5B). Although there were no significant changes in H3K27me3-binding peak numbers and associated genes number (Additional file 1: Fig. S3B) after *Rif1* knockdown, the average H3K27me3 intensity on all peaks was significantly down-regulated with *Rif1*-KD (Fig. 5C). Interestingly, the H3K27me3 decreases occurred more frequently in the promoter regions (Additional file 1: Fig. S3C), indicating that *Rif1*-KD-induced H3K27me3 reduction may have a greater transcriptional effect. Additionally, ontology analysis showed that the genes with reduced H3K27me3 level had higher enrichments on mESCs differentiation and development terms, which was consistent with the function of *Rif1* (Additional file 1: Fig. S3D). Overall, 76.45% of RIF1 bound genes were also occupied by H3K27me3 (Fig. 5D). The transcription levels of 363 of these genes were up-regulated and H3K27me3 occupancy on their promoters was down-regulated by *Rif1* knockdown (Fig. 5D). These overlapping genes showed an enrichment on embryonic morphogenesis and stem cell differentiation, which was consistent with the RNA-seq and RIF1 ChIP-seq data (Fig. 5E).

As ChIP-seq data came from *Rif1*-KD mESCs, we compared the *Rif1*-KO and KD RNA-seq data. First, we performed PCA analysis and found that KO and KD samples had very close coordinates (Additional file 1: Fig. S3E). Next, we calculated the *Pearson* correlations of these transcriptomes and observed that the coefficients between KO and KD samples were greater than 0.9 (Additional file 1: Fig. S3F). We further intersected the differentially expressed genes (DEGs) of *Rif1*-KD and KO data and found that most of the DEGs (1625 out of 1834) in *Rif1*-KO were also mis-regulated in KD results (Additional file 1: Fig. S3G-H). The gene ontology analysis of *Rif1*-KD also had very similar enriched terms compared to *Rif1*-KO (Fig. 5E and additional file 1: Fig. S3I). All these results demonstrated the similarity between *Rif1*-KD and KO RNA-seq data.

For the differentiation genes that were up-regulated by *Rif1* knockout, the occupancy of both H3K27me3 within ± 5 kilobase (kb) windows of the peak center decreased in *Rif1*-KD mESCs (Fig. 5F). In addition, the occupancy of EZH2 and H3K27me3 on the promoter regions of mesendodermal genes also declined in response to *Rif1* deficiency (Fig. 5G and additional file 1: Fig. S4A), which was confirmed by ChIP-qPCR (Fig. 5H, I and additional file 1: Fig. S4B). The co-immunoprecipitation assay showed that RIF1 interacted with EZH2 in E14 mESCs [30]. Bivalent genes are important for embryonic stem cell differentiation and are regulated by the PRC1 and PRC2 complexes [38]. To check how many RIF1-regulated genes are bivalent, we first downloaded the full list of bivalent genes in mESCs from the published data [39]. We found that more than 25% (1063 out of 4239) of RIF1-occupied genes overlapped with the mESCs bivalent genes (Additional file 1: Fig. S4C and additional file 3: Table S3). Notably, de novo motif discovery revealed that the binding motifs of *Prdm15*, *Foxd1*, *Sox17*, *Hoxa2* and *Pou3f3*, which are key regulators of the process of mESCs development and differentiation, were enrich ed on the altered H3K27me3 binding regions of *Rif1* knockdown (Fig. 5J).

Collectively, these results demonstrate that RIF1 interacts with EZH2 and that RIF1 recruits EZH2 to RIF1-binding sites on the promoter regions of mesendodermal genes, which promotes the trimethylation of H3K27 and represses mesendodermal gene expression.

### *Rif1* Deficiency Promotes Mesendodermal Differentiation by Activating ERK1/2 Activities

**Fig. 6:**
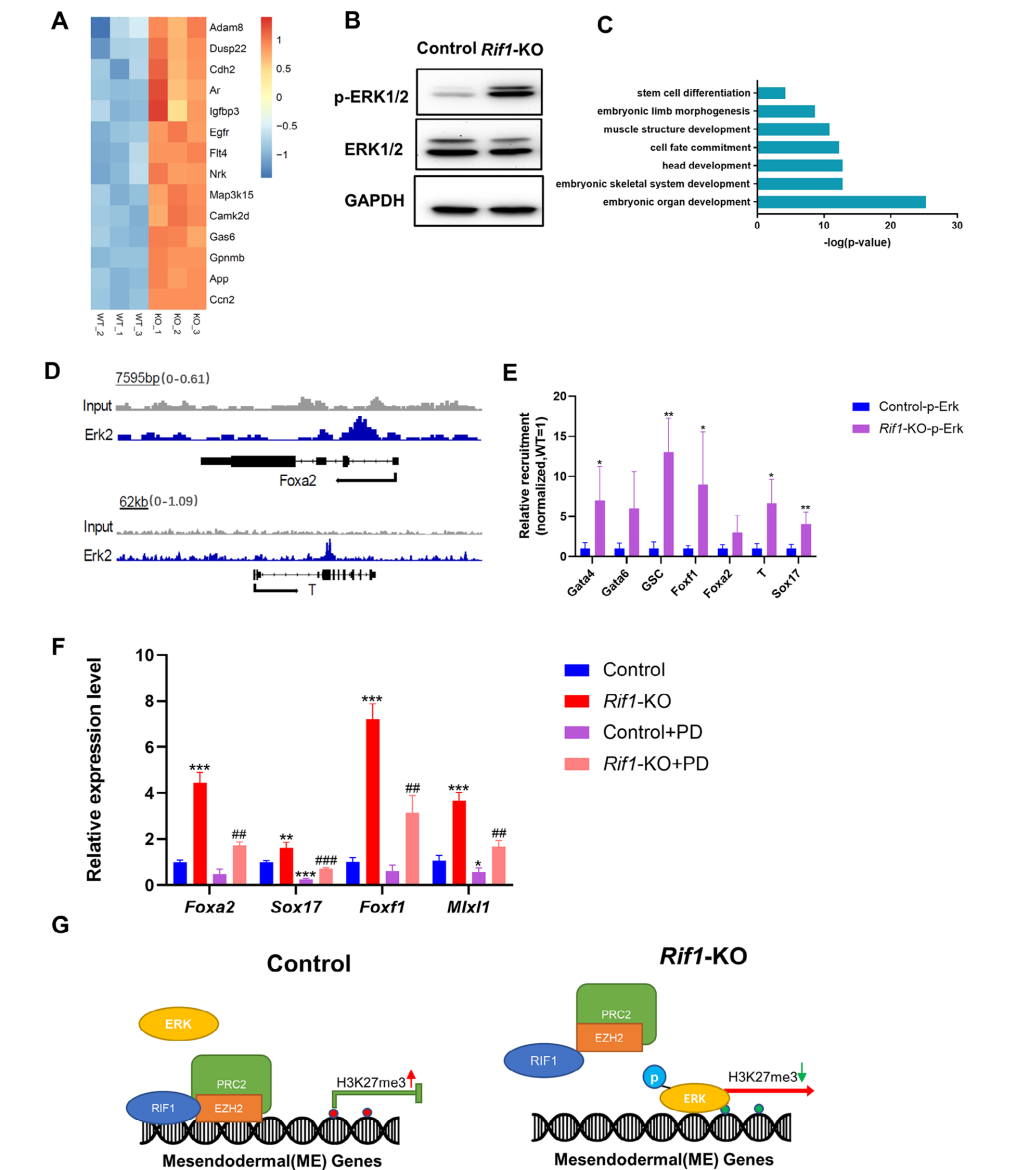
***Rif1*-KO promotes mesendodermal differentiation by activating ERK1/2 activities**. **(A)** The heatmap of up-regulated genes that positively regulate MAPK cascade. **(B)** The protein levels of p-ERK1/2 and ERK1/2 were valuated via Western blot. **(C)** GO analysis for biological processes associated with genes occupied by ERK2. **(D)** Genome tracks showing ERK2 occupancy near lineage-specific markers for endoderm (*Foxa2*) and mesoderm (*Brachyury*). **(E)** The binding of promoter sequences for mesendodermal genes to p-ERK1/2 was evaluated in control and *Rif1*-KO mESCs via ChIP-qPCR; results were normalized to control cells (n = 3). *p < 0.05; **p < 0.01 compared with control; t test. **(F)** The relative mRNA levels of mesendodermal genes were evaluated in control and *Rif1*-KO mESCs that had been treated with or without PD0325901(1 μm) on day 4 of differentiation compared with control (n = 3). *p < 0.05; **p < 0.01; ***p < 0.001 compared with control (PD-); #*p* < 0.05; ##*p* < 0.01 compared with *Rif1*-KO (PD-); one-way analysis of variance. **(G)** Model showing that RIF1 plays an important role in mESCs for suppression of mesendodermal differentiation. RIF1 also impedes mesendodermal differentiation by recruiting PRC2, which leads to the displacement of H3K27me3 and gene silencing, as well as inhibiting ERK1/2 signaling. Data were collected from three independent replicates and are shown as means ± SEM.

The mesendodermal specification of mESCs is mediated, in part, by the activation of ERK1/2 signaling [15], and our RNA-seq analysis has demonstrated that *Rif1* knockout positively regulated MAPK signaling (Fig. 6A). And the overlapping genes that altered in *Rif1*-KO and *Erk*-KO mESCs are differentiation related genes (Additional file 1: Fig. S5A, B). In addition, loss of *Rif1* was associated with the increased protein levels of p-ERK1/2 (Fig. 6B).

To explore if *Rif1*-KO regulates the expression of mesendodermal genes through p-ERK1/2 activation, we analyzed previously published ChIP-seq data on ERK2 [12]. GO analysis of ERK2-bound genes showed an enrichment on embryonic organ development and stem cell differentiation (Fig. 6C). The genome sessions showed that ERK2 occupied genomic regions of mesendodermal genes (Fig. 6D). ChIP-qPCR analysis confirmed that the p-ERK1/2 binding to mesendodermal gene promoters was significantly greater in *Rif1*-KO mESCs than in control (Fig. 6E). Mesendodermal gene expression increased on day 4 of EB differentiation in *Rif1*-KO mESCs while decreased significantly when treated with the p-ERK1/2 inhibitor, PD0325901 (Fig. 6F).

Thus, the mesendodermal specification of mESCs regulated by *Rif1* deficiency is mediated, as least partially, by the activation of ERK1/2 signaling.

## Discussion

*Rif1* is highly expressed in ESCs and testes but is not detectable in MEF and other somatic tissues [31]. *Rif1* deficiency results in embryogenesis defects and impairs differentiation of ESCs toward the neural lineage, but the study evaluated lineage-specific genes in day 10 EBs, which is a later stage of EB differentiation, so whether *Rif1* affects mesoderm and endoderm differentiation remains unclear [29]. However, whether *Rif1* regulates the early differentiation of mESCs is unknown. Here, our data demonstrated that *Rif1* maintains the self-renewal and pluripotent state of mESCs and represses the expression of lineage-specific genes (Fig. 6H). We also showed that RIF1 targets the promoters of lineage-specific genes with PRC2 complex to repress their expression in mESCs. Thus, *Rif1* appears to be a key determinant regulator of cell fate and lineage specification in mESCs.

RIF1 was first identified as a telomere-associated protein in budding yeast that negatively regulates telomere length [16] but in fact it has more functions. Previous reports have shown that RIF1 is a key factor in DNA damage response and DNA replication timing [20–22, 24–28]. In mammals, RIF1 expression is significantly higher in embryonic stem cells compared to differentiated cells, indicating that it may play an important role in mESCs. RIF1 was also identified as a key factor for self-renewal of mESCs by Genome-wide RNAi screen [40] and it is one of the Nanog-associated partners, meaning that RIF1 is in the pluripotent interaction networks [34]. Consistent with previous studies, our data also showed that RIF1 is a transcriptional suppressor and affects self-renewal and pluripotency [29]. In addition, we also proved that RIF1 regulates the differentiation of mESCs. Previous studies showed that the neural lineage but not the endoderm or mesoderm lineage was affected and the teratoma couldn’t be formed by *Rif1* knockdown in mESCs [29] while our findings demonstrated that *Rif1*-KO promotes mesendoderm differentiation in vitro and vivo. A possible explanation may be that the proliferation and self-renewal abilities of mESCs and the capacity to form embryoid bodies and teratomas increased under 2i medium, because the 2i culture condition is able to better mimic the ground-state pluripotency of the developing embryo, resulting in a more homogeneous pluripotent state compared to the serum condition. The epigenome and transcriptome of 2i or serum cultured ESCs are markedly different [41]. More specifically, 2i ESCs show an identity close to the early blastocyst cells of the inner cell mass (ICM) or even earlier stages, while serum ESCs more resemble the later stage cells [14, 42, 43]. Collectively, our observations demonstrated that RIF1 plays an important role in cell identity and differentiation of mESCs.

Stem cell pluripotency and cell lineage specification during development are regulated by PRC2, which is essential for repressive H3K27 methylation [13, 44]. PRC2 catalyzes the di- and trimethylation of H3K27 by its subunit EZH2 [5, 45]. The deficiency of EZH2 reduces the pluripotency and promotes the spontaneous differentiation of human embryonic stem cells (hESCs) toward the mesendodermal fate [7]; RIF1 was identified as a novel epigenetic modifier involved in transcriptional regulation in mice by ENU mutagenesis screen [46]. RIF1 promotes the establishment of multiple repressive chromatin marks, including H3K9me3, H3K27me3 and DNA methylation in mESCs by interacting with HMTs [29, 30]. Here, we showed that *Rif1* knockout up-regulates the mesendodermal genes expression and induces the mesendodermal specification of mESCs. In terms of mechanism, RIF1 interacts with EZH2 and promotes H3K27me3 on the promoters of numerous mesendodermal genes. And RIF1 cooperates with a series of key factors, such as c-Myc and Sox family genes, to regulate embryonic stem cell differentiation. Collectively, our observations revealed that RIF1 represses differentiation in mESCs by recruiting the histone methyltransferase (HMT).

Our results also indicated that *Rif1* deficiency positively regulates the MAPK cascade. The induction of mesendoderm differentiation was accompanied by an increase in phosphorylation of ERK, which is a critical signaling for ESC self-renewal. Our data are consistent with previous reports that ERK signaling is essential for the activation of differentiation genes [12]. ERK1/2 signaling is also involved in the transition between the pluripotency state of mESCs and epiblast stem cells (EpiSCs) [47]. The role of ERK in lineage commitment in mESCs is a recognized feature, and it is also essential for the maintenance of self-renewal in hESCs [48]. ERK2 is also associated with transcriptional regulation in hESCs [48]. In addition, ERK1/2 activity regulates the PRC2 occupancy and promotes RNAPII phosphorylation on the promotors of developmental genes in mESCs [15]. Collectively, our data showed that increased MAPK pathway activity and ERK signaling collaborated with RIF1-regulated H3K27me3 in priming the cells differentiating more efficiently toward the endoderm and mesoderm lineages in mESCs.

## Conclusion

In summary, the results presented here showed that RIF1 maintains stem cell identity in mESCs by silencing mesendodermal gene expression through PRC2-induced H3K27me3 of functional genes’ promoters and inhibiting the MAPK signaling.

## Electronic Supplementary Material

Below is the link to the electronic supplementary material.

Supplementary Material 1

Supplementary Material 2

Supplementary Material 3

Supplementary Material 4

## Data Availability

*Rif1*-KO RNAseq data were uploaded to Gene Expression Omnibus (GEO) with accession number GSE224763. The wild type and *Rif1*-KD H3K27me3 ChIP-seq data (peak and bigWig files) were downloaded from GEO GSE98256. The wild type EZH2 ChIP-seq data were downloaded from GEO GSE74330. The ERK2 ChIP-seq data were downloaded from GEO SRP028688. *Rif1*-KD RNAseq data were from GSE98256.

## Code Availability

Noting to declare.

## References

[1] Croce LD, Helin K (2013). Transcriptional regulation by polycomb group proteins. Nature Structural & Molecular Biology.

[2] Tong IL (2006). Control of developmental regulators by polycomb in human embryonic stem cells. Cell.

[3] Margueron R (2008). Ezh1 and Ezh2 maintain repressive chromatin through different mechanisms. Molecular Cell.

[4] Piunti A, Shilatifard A (2016). Epigenetic balance of gene expression by polycomb and COMPASS families. Science.

[5] Ru C (2002). Role of histone H3 lysine 27 methylation in polycomb-group silencing. Science.

[6] O"Carroll D (2001). The polycomb-group GeneEzh2 is required for early mouse development. Molecular & Cellular Biology.

[7] Adam (2016). Deletion of the polycomb-group protein EZH2 leads to compromised Self-Renewal and differentiation defects in human embryonic stem cells. Cell Reports.

[8] Mu W (2018). EZH2 variants differentially regulate polycomb repressive complex 2 in histone methylation and cell differentiation. Epigenetics & Chromatin.

[9] Shaul YD, Seger R (2007). The MEK/ERK cascade: From signaling specificity to diverse functions. Biochimica Et Biophysica Acta Molecular Cell Research.

[10] Pearson G (2001). Mitogen-activated protein (MAP) kinase pathways: Regulation and physiological functions. Endocrine Reviews.

[11] Burdon T (1999). Suppression of SHP-2 and ERK signalling promotes self-renewal of mouse embryonic stem cells. Developmental biology.

[12] Chen, H. (2015). Erk signaling is indispensable for genomic stability and self-renewal of mouse embryonic stem cells. Proceedings of the National Academy of Sciences of the United States of America, : p.5936–43. 10.1073/pnas.1516319112 PMC4640739 26483458

[13] Shen X (2008). EZH1 mediates methylation on histone H3 lysine 27 and complements EZH2 in maintaining stem cell identity and executing pluripotency. Molecular Cell.

[14] Ying QL (2008). The ground state of embryonic stem cell self-renewal. Nature.

[15] Tee WW (2014). Erk1/2 activity promotes chromatin features and RNAPII phosphorylation at Developmental Promoters in Mouse ESCs. Cell.

[16] Hardy CF, Sussel L, Shore D (1992). A RAP1-interacting protein involved in transcriptional silencing and telomere length regulation. Genes & Development.

[17] Teixeira MT (2004). Telomere length homeostasis is achieved via a switch between telomerase- extendible and -Nonextendible States. Cell.

[18] Levy DL, Blackburn EH (2004). Counting of Rif1p and Rif2p on Saccharomyces cerevisiae telomeres regulates telomere length. Molecular and cellular biology.

[19] Buonomo, S., Heterochromatin, D. N. A., & Rif1. (2010). replication and. Experimental Cell Research, 316(12): p. 1907–1913. 10.1016/j.yexcr.2010.03.015 20347809

[20] Chapman JR (2013). RIF1 is essential for 53BP1-dependent nonhomologous end joining and suppression of DNA double-strand break resection. Molecular Cell.

[21] Fradet-Turcotte A (2013). 53BP1 is a reader of the DNA-damage-induced H2A lys 15 ubiquitin mark. Nature.

[22] Silverman (2004). Human Rif1, ortholog of a yeast telomeric protein, is regulated by ATM and 53BP1 and functions in the S-phase checkpoint. Genes & Development.

[23] Xu D (2010). Rif1 provides a new DNA-binding interface for the Bloom syndrome complex to maintain normal replication. The EMBO Journal.

[24] Zimmermann M (2013). 53BP1 regulates DSB repair using Rif1 to Control 5′ end resection. Science.

[25] Zachary M (2018). 53BP1–RIF1–shieldin counteracts DSB resection through CST- and Polα-dependent fill-in. Nature.

[26] Cornacchia D (2012). Mouse Rif1 is a key regulator of the replication-timing programme in mammalian cells. The EMBO Journal.

[27] Hayano M (2012). Rif1 is a global regulator of timing of replication origin firing in fission yeast. Genes & Development.

[28] Yamazaki S (2012). Rif1 regulates the replication timing domains on the human genome. The EMBO Journal.

[29] Jiameng (2014). Rif1 maintains telomere length homeostasis of ESCs by mediating heterochromatin silencing. Developmental Cell.

[30] Li, P. Rif1 promotes a repressive chromatin state to safeguard against endogenous retrovirus activation. Nuclc Acids Research, 2017(22): p.12723–12738. 10.1093/nar/gkx884 PMC5727408 29040764

[31] Adams IR, Mclaren A (2004). Identification and characterisation of mRif1: A mouse telomere-associated protein highly expressed in germ cells and embryo-derived pluripotent stem cells. Developmental Dynamics.

[32] G., et al., A genome-wide RNAi screen identifies a new transcriptional module required for self-renewal. Genes & Development, 23(7):837–48. 10.1101/gad.1769609 PMC2666338 19339689

[33] Loh YH (2006). The Oct4 and nanog transcription network regulates pluripotency in mouse embryonic stem cells. Nature Genetics.

[34] Jianlong (2006). A protein interaction network for pluripotency of embryonic stem cells. Nature, 444(7117). 10.1038/nature05284 17093407

[35] Buonomo (2009). Mammalian Rif1 contributes to replication stress survival and homology-directed repair. Journal of Cell Biology.

[36] Berg DLCVD (2010). An Oct4-Centered protein Interaction Network in embryonic stem cells. Cell Stem Cell.

[37] Wang J (2006). A protein interaction network for pluripotency of embryonic stem cells. Nature.

[38] Macrae, T. A., Fothergill-Robinson, J., & Ramalho-Santos, M. (2022). Regulation, functions and transmission of bivalent chromatin during mammalian development. Nature Reviews Molecular Cell Biology, : p.1–21. 10.1038/s41580-022-00518-2 36028557

[39] Ku M (2008). Genomewide analysis of PRC1 and PRC2 occupancy identifies two classes of Bivalent Domains. PLoS Genetics.

[40] Hu G (2015). A genome-wide RNAi screen identifies a new transcriptional module required for self-renewal. Genes & Development.

[41] Marks H (2012). The transcriptional and epigenomic foundations of ground state pluripotency. Cell.

[42] Schlesinger, S., Meshorer, E., & Chromatin, O., Epigenetic Plasticity, and Nuclear Organization in Pluripotency. Developmental Cell, 2019 Jan 28;48(2):p.135–150. 10.1016/j.devcel.2019.01.003 30695696

[43] Marks, S. (2014 Mar). Transcription regulation and chromatin structure in the pluripotent ground state. *Biochimica et Biophysica Acta*, *1839*(3), p129–p137. 10.1016/j.bbagrm.2013.09.005 24096207

[44] Riising E (2014). Gene silencing triggers polycomb repressive complex 2 recruitment to CpG islands genome wide. Molecular Cell.

[45] Faust C (1998). The polycomb-group gene eed is required for normal morphogenetic movements during gastrulation in the mouse embryo. Development.

[46] Daxinger L (2013). An ENU mutagenesis screen identifies novel and known genes involved in epigenetic processes in the mouse. Genome Biology.

[47] Greber B (2010). Conserved and divergent roles of FGF signaling in mouse epiblast stem cells and human embryonic stem cells. Cell Stem Cell.

[48] Göke J (2013). Genome-wide kinase-chromatin interactions reveal the Regulatory Network of ERK Signaling in Human Embryonic Stem cells. Molecular Cell.

