# Supplementary material for: Rif1 Regulates Self-Renewal and Impedes Mesendodermal Differentiation of Mouse Embryonic Stem Cells
Source: Stem Cell Rev Rep. 2023 Mar 27;19(5):1540–53. doi: 10.1007/s12015-023-10525-1 (PMC10366267; doi:10.1007/s12015-023-10525-1)
Supplement: Supplementary file 1 — Supplementary Material 1 [file 12015_2023_10525_MOESM1_ESM.docx]

| 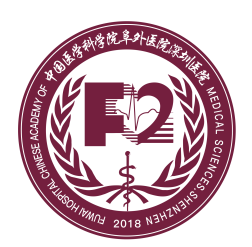 | Chinese Academy of Medical Sciences, Fuwai Hospital |
| --- | --- |

State Key Laboratory of Cardiovascular Disease

Fuwai Hospital

National Center for Cardiovascular Diseases

CAMS, Beijing 100037, China

Dear Stem Cell Reviews and Reports Editor,

It is our pleasure to submit a revised version of our manuscript entitled “***Rif1* regulates self-renewal and impedes mesendodermal differentiation of mouse embryonic stem cells**” for further consideration by *Stem Cell Reviews and Reports*. We are deeply grateful for the immensely insightful reviewer comments.

In summary, the reviewers’ concerns were mainly focused on the following aspects:

1. **Limitations in experiments or data analyses**

To fully support the conclusion that Rif1 regulates mESCs differentiation, we performed additional experiments, including evaluating the OCT4 protein degradation induced by RIF1-KO (**new Fig. S1A**), checking both mRNA and protein levels of RIF1 during differentiation **(new Fig. 3B)** and the molecular signatures of the mesendodermal markers (**new Fig. S3G and Fig. S2D**)

We also improved our analyses of previously generated data, including comprehensively re-analyzing Rif1 and H3K27me3 ChIP-seq data (**revised Fig. 4 and 5, new Fig. S2E-G, S4A**), assessing the similarity between Rif1-KO and KO RNA-seq data (**new Fig. S3E-I**), quantifying the three-germ layer composition in teratomas **(new Fig. S2C),** and performing proper statistical tests and labelling *P*-values (**revised Fig. 3A**).

1. **Manuscript editing and discussion**

We thoughtfully edited the text of our manuscript, with the assistance from a native English speaker colleague. As the reviewer suggested, we have corrected the inproper words, sentences, and typos in Figure legends and Methods sections, as well as in the main text. To improve the quality of the figures, we replaced or added more representative images **(revised Fig 3D, 5B, and new Fig. S4A)**. We also added the missing references and clarified all the sequencing data we used in the Data availability section.

According to the reviewers’ comments, we have highlighted the binding motifs of Rif1 and its partners and discussed their roles in embryonic stem cell differentiation. We also provided detailed information on the 2i medium and compared it to normal serum medium.

In this resubmission, we provide a detailed point-to-point response letter that addresses all concerns raised. Additionally, we also submit our revised manuscript and supplemental material (major text revisions are highlighted in yellow), which have been significantly improved thanks to the valuable advice from the reviewers. Thank you for your reconsideration, and we look forward to the comments and suggestions from you and reviewers.

Sincerely,


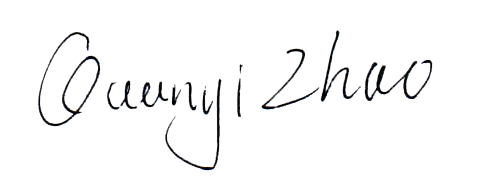


Quanyi Zhao, Ph.D.

State Key Laboratory of Cardiovascular Disease, Fuwai Hospital

Chinese Academy of Medical Sciences, Beijing 100037, China

Phone: +86 156 1804 6819;

Email: [zhaoquanyi@fuwaihospital.org](mailto:zhaoquanyi@fuwaihospital.org)
