# Supplementary material for: Rif1 Regulates Self-Renewal and Impedes Mesendodermal Differentiation of Mouse Embryonic Stem Cells
Source: Stem Cell Rev Rep. 2023 Mar 27;19(5):1540–53. doi: 10.1007/s12015-023-10525-1 (PMC10366267; doi:10.1007/s12015-023-10525-1)
Supplement: Supplementary file 3 — Supplementary Material 3 [file 12015_2023_10525_MOESM3_ESM.docx]

**Rif1 regulates self-renewal and impedes mesendodermal differentiation of mouse embryonic stem cells**

Zhao *et al*.

**Supplemental Figures and Legends**

**Supplemental Figure 1**

**Supplemental Figure 2**

**Supplemental Figure 3**

**Supplemental Figure 4**

**Supplemental Figure 5**

**Supplemental Table 1 Primer sets used in this study.**


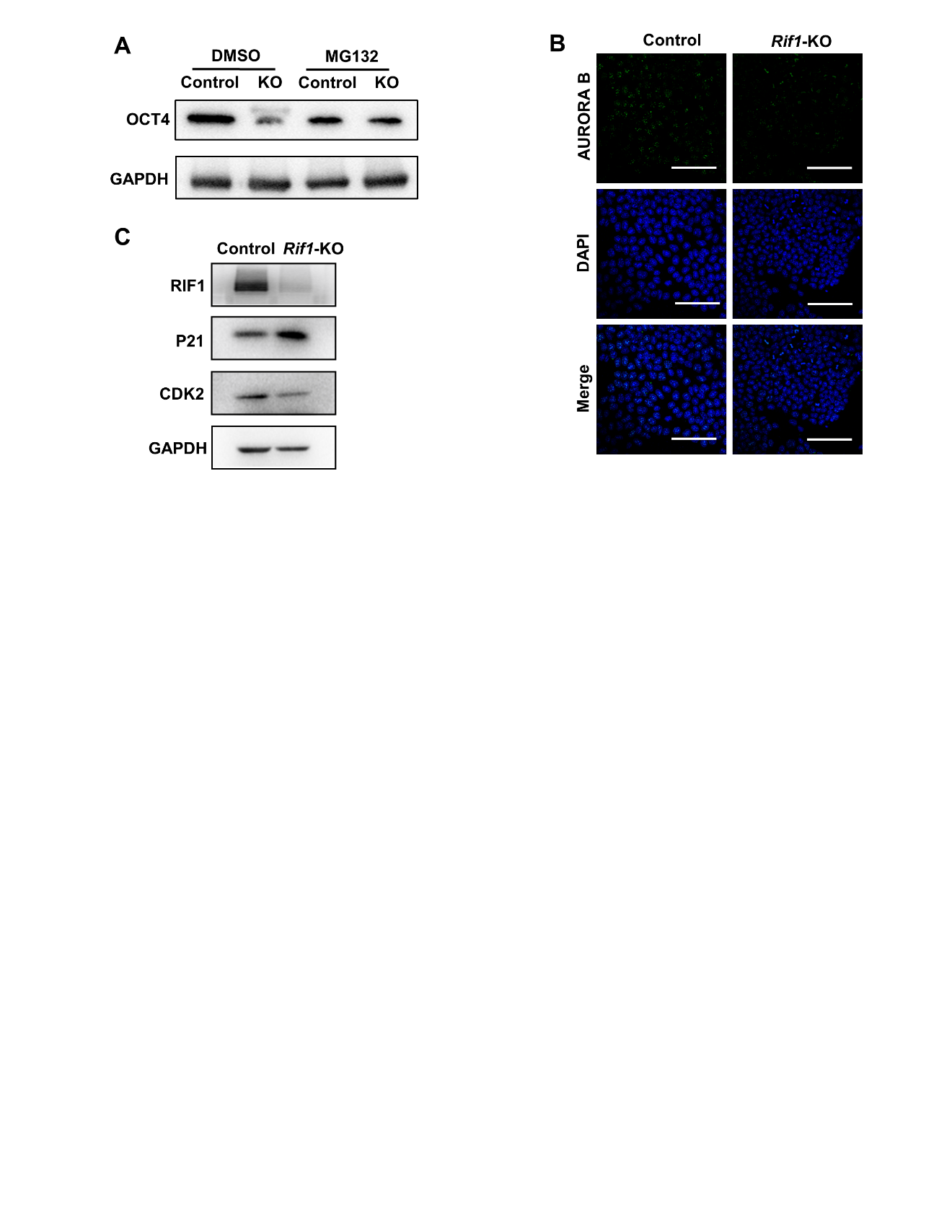


**Supplemental Figure 1**. **Depletion of Rif1 impairs mESCs proliferation and cell cycle.**

**A.** Control and Rif1-KO mESCs were treated with or without

proteasome inhibitor MG132 (10 mM) for 6 hours, and the levels of Oct4 proteins were determined by immunoblotting. **B.** Control and *Rif1*-KO mESCs were immunofluorescently stained for AURORA B expression, and nuclei were counter stained with DAPI (blue). **C.** The protein levels of CDK2 and P21 were evaluated via Western blot.


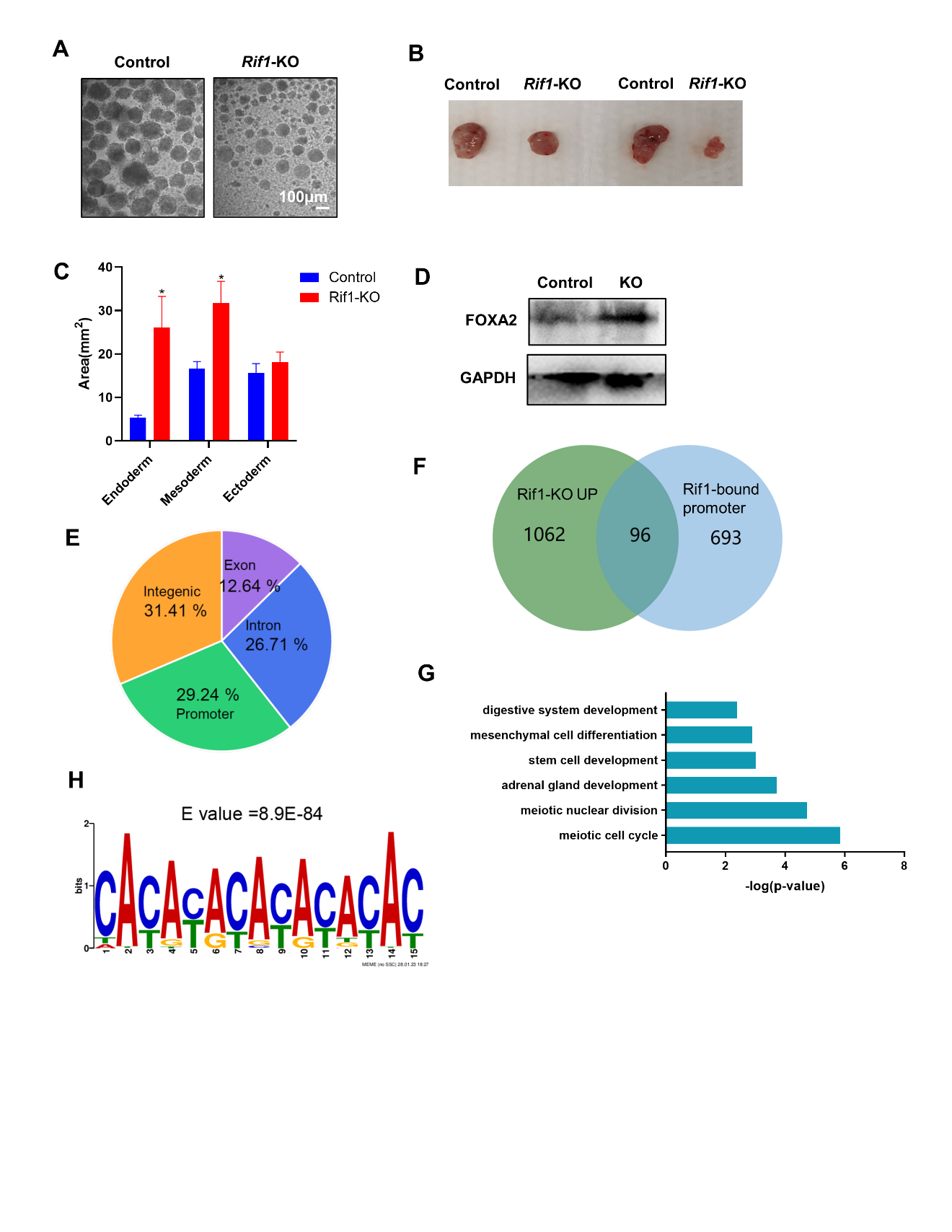


**Supplemental Figure 2**. **RIF1 deficiency promotes mesendoderm differentiation.**

**A.** The morphology of EB derived from control and Rif1-KO mESCs at Day 3. Scale bars, 100 μm. **B.** Morphology of teratomas from control and Rif1-KO mESCs**. C.** The Histogram showing the area of three germ layers. **D.** Foxa2 protein level was evaluated in teratomas via Western blot. **E.** The pie chart shows the genomic distribution of RIF1 binding sites for these 381 genes. **F.** Venn diagram showing the overlap between genes upregulated by RIF1 deletion with FC>0.58 and genes whose promoter regions are occupied by RIF1(upper panel). **G.** GO analysis for biological processes associated with overlapped genes (lower panel). **H.** The motif of the RIF1 binding regions in wild type sample.


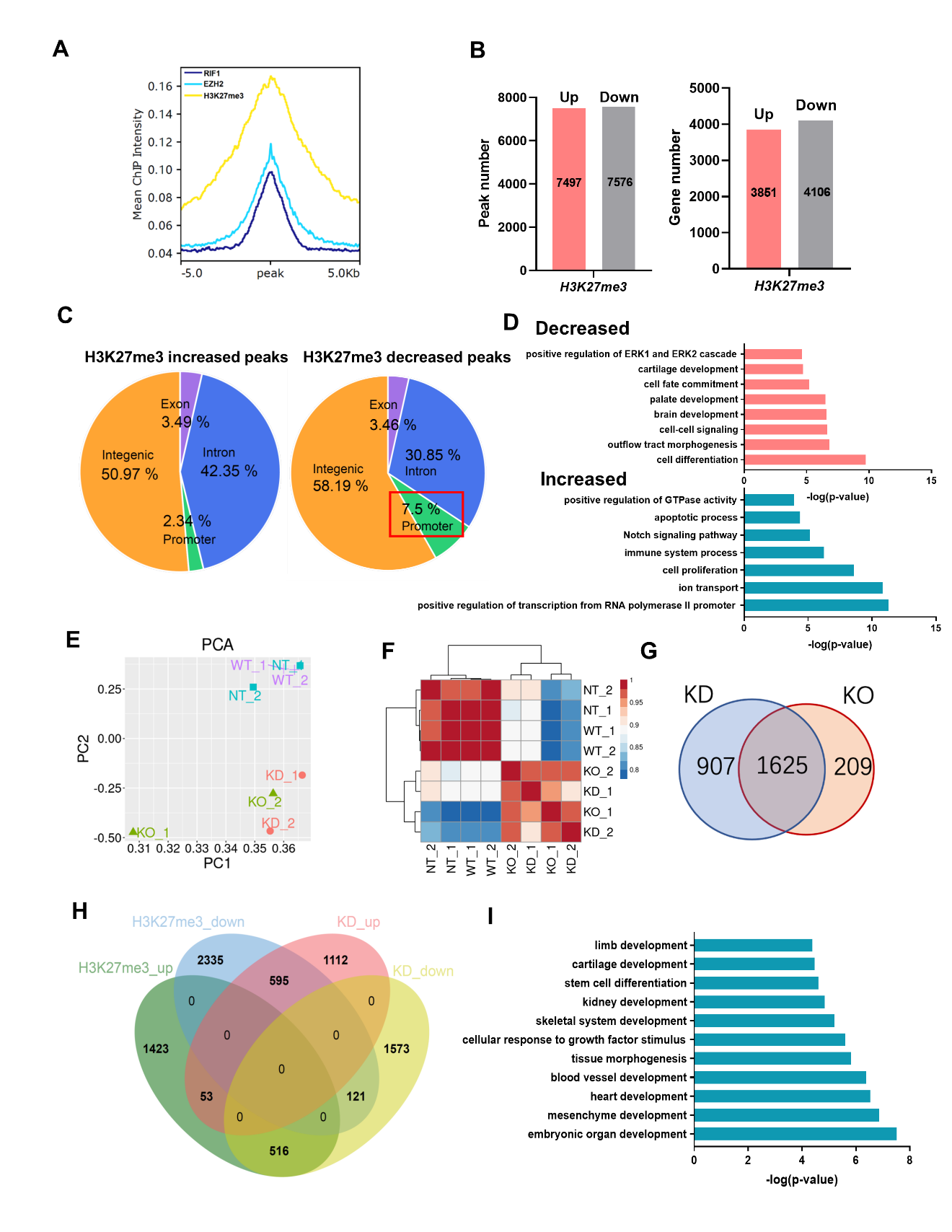


**Supplemental Figure 3**. **RIF1 deficiency activates differentiation and development-related genes expression**

**A.** ChIP-seq signals of H3K27me3, RIF1 and EZH2 near RIF1 peak center. **B.** The histogram shows the number of peaks and genes that H3K27me3 occupies changes. **C.** Pie chart showing the genomic distribution of H3K27me3 binding sites. **D.** GO analysis for biological processes associated with genes that have decreased and increased occupancy of H3K27me3 in *Rif1*-KD mESCs. **E.** The PCA analysis with Rif1-KO and KD transcriptomes. **F.** Pearson correlation coefficients of transcriptomes. **G.** Overlap between genes altered by Rif1 knockout and knockdown. **H.** Venn diagram showing the overlap between genes differentially regulated by Rif1 knockdown with FC > 1.5 and genes that have different occupancy of H3K27me3. **I.** GO analysis for biological processes associated with overlapped genes between genes upregulated by Rif1 knockdown and genes that have decreased occupancy of H3K27me3.

`


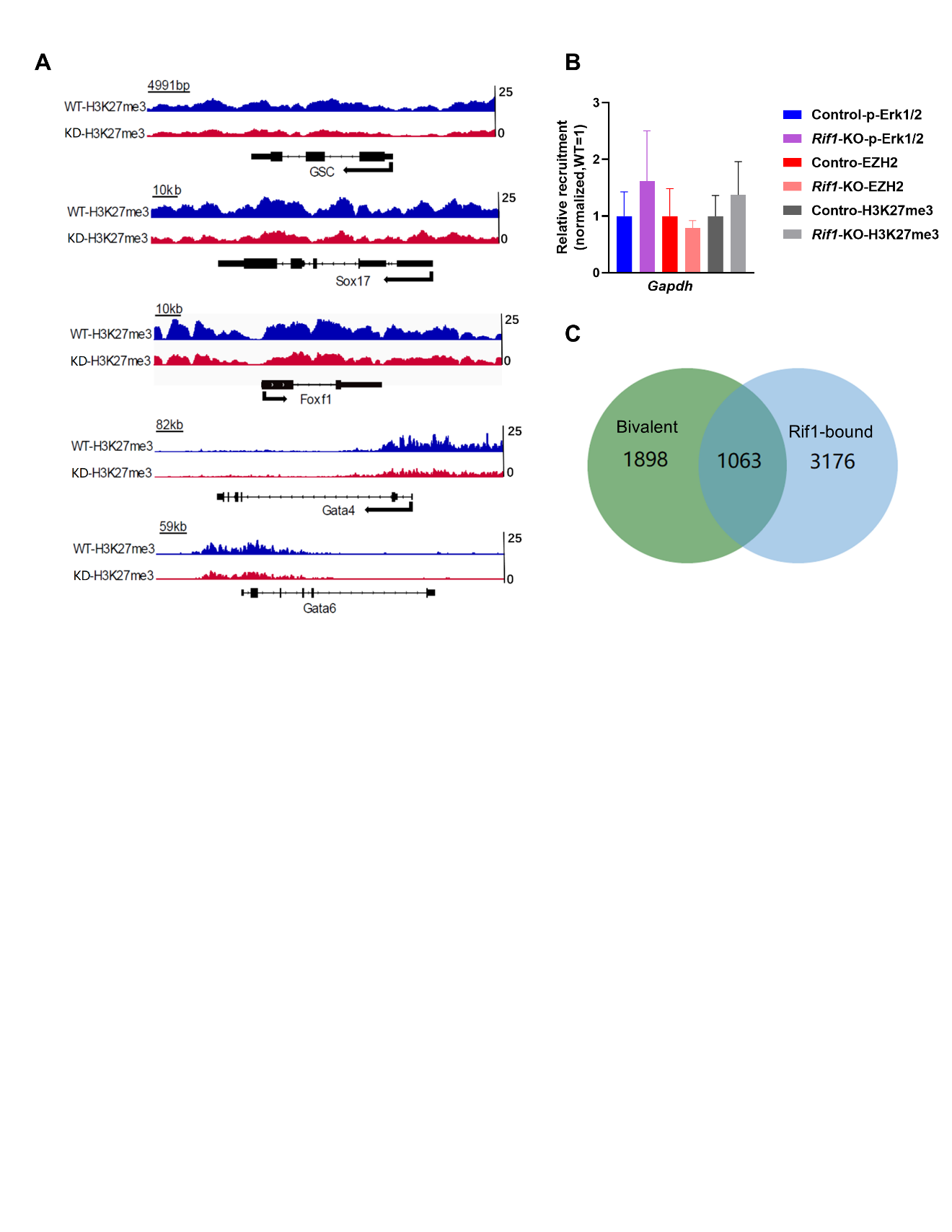


**Supplemental Figure 4**. **RIF1 deficiency activates differentiation and development-related genes expression**

**A.** Genome tracks showing differential occupancy by H3K27me3 near lineage-specific markers for endoderm and mesoderm. **B.** The binding of promoter sequences for *Gapdh* to p-ERK1/2, EZH2, H3K27me3 was evaluated in control and *Rif1*-KO mESCs via ChIP-qPCR (n = 3). **C**. The overlap between bivalent genes and RIF1-binding genes.


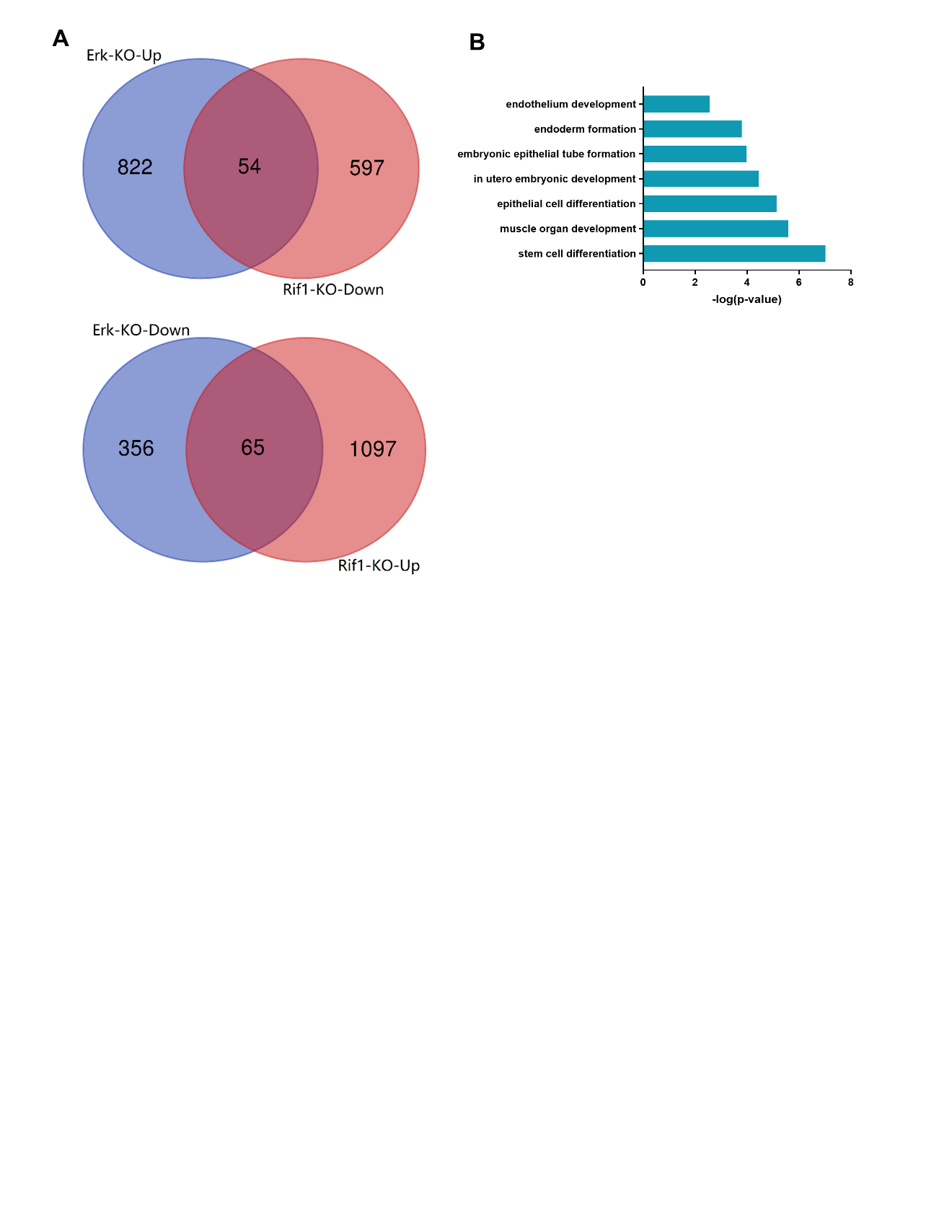


**Supplemental Figure 5. The overlapping genes altered in Rif1-KO and Erk-KO cells are differentiation-related genes.**

**A.** Venn diagrams showing overlap between *Rif1*-KO DEGs and *Erk*-KO DEGs in mESCs. **B.** GO analysis for biological processes associated with the genes that are both up regulated in *Rif1*-KO RNA-seq and down regulated in *Erk*-KO RNA-seq.

**Supplemental Table 1. Primer sets used in this study.**

| mqRT-Rif1-F | AGTCATTGGTTTCTTCATTGTAGGC |
| --- | --- |
| mqRT-Rif1-R | GTGCTGAGTGAACAACTAATGGTA |
| mqRT-Fgf5-F | GAAATATTTGCTGTGTCTCAGGG |
| mqRT-Fgf5-R | TAAATTTGGCACTTGCATGG |
| mqRT-Sox17-F | CTTTATGGTGTGGGCCAAAG |
| mqRT-Sox17-R | TTCCAAGACTTGCCTAGCATC |
| mqRT-Foxa2-F | CCATCAGCCCCACAAAATG |
| mqRT-Foxa2-R | CCAAGCTGCCTGGCATG |
| mqRT-T-F | CATCGGAACAGCTCTCCAACCTAT |
| mqRT-T-R | GTGGGCTGGCGTTATGACTCA |
| mqRT-Pax6-F | TAACGGAGAAGACTCGGATGAAGC |
| mqRT-Pax6-R | CGGGCAAACACATCTGGATAATGG |
| mqRT-Otx2-F | CAGCGTCCATCTCCCCAC |
| mqRT-Otx2-R | GTTGAGCCAGCATAGCCTTG |
| mqRT-Foxf1-F | GCATCCCTCGGTATCACTCAC |
| mqRT-Foxf1-R | ATCCTCCGCCTGTTGTATGC |
| mqRT-Gapdh-F | CCCCAATGTGTCCGTCGTG |
| mqRT-Gapdh-R | TGCCTGCTTCACCACCTTCT |
| mqRT-Mixl1-F | CGCTCCCTCAGTAACAACGC |
| mqRT-Mixl1-R | GCTGCCACAGACTTCCAAATG |
| mqRT-Gsc-F | ACCATCTTCACCGATGAGCAGC |
| mqRT-Gsc-R | CTTGGCTCGGCGGTTCTTAAAC |
| mqRT-Esrrb-F | CAAGAGAACCATTCAAGGCAACA |
| mqRT-Esrrb-R | CATCCCCACTTTGAGGCATTT |
| mqRT-Oct4-F | GAAGCAGAAGAGGATCACCTTG |
| mqRT-Oct4-R | TTCTTAAGGCTGAGCTGCAAG |
| mqRT-Nanog-F | CCTCAGCCTCCAGCAGATGC |
| mqRT-Nanog-R | CCGCTTGCACTTCACCCTTTG |
| mqRT-Klf4-F | AGGAACTCTCTCACATGAAGCG |
| mqRT-Klf4-R | GGTCGTTGAACTCCTCGGTC |
| mqRT-Sox2-F | CGGCACAGATGCAACCGAT |
| mqRT-Sox2-R | CCGTTCATGTAGGTCTGCG |
| ChIP-qPCR-Foxa2_F | TAGGTGAGAGGAAGCCAGGG |
| ChIP-qPCR-Foxa2_R | GACTCCCCGTGGACGTAAAG |
| ChIP-qPCR-Gata4_F | GAGAGGGTCAGGAGGCACTA |
| ChIP-qPCR-Gata4_R | GGCTCGTGGGAAGGAGAAAA |
| ChIP-qPCR-Sox17_F | CTCTGAGAACACCGAGCGTC |
| ChIP-qPCR-Sox17_R | GTGAAAGGTGCCAATCGACC |
| ChIP-qPCR-Gata6_F | GTGTCCGGTCCTTCGCTTTA |
| ChIP-qPCR-Gata6_R | GCGCTGTGATAACTCGGAGA |
| ChIP-qPCR-T_F | CTCCGCAGAGTGACCCTTTT |
| ChIP-qPCR-T_R | GTACTGCAGGCTCTTCCCTG |
| ChIP-qPCR-GSC_F | GGTAGAAGGCGCCGTAGTC |
| ChIP-qPCR-GSC_R | TGGTCTTCCCGGCTCTACAC |
| ChIP-qPCR-Foxf1_F | CTGTCGCTCAACGAGTGCTT |
| ChIP-qPCR-Foxf1_R | ACATAAACTCGCTAGCCGGA |
| ChIP-qPCR-Gapdh_F | GTGTGGGCTCCGAACTGATA |
| ChIP-qPCR-Gapdh_R | CCCTGAGTCCTATCCTGGGAA |
| mqRT_Adam8_F | TGCTCAGCGTCTTATGGACAC |
| mqRT_Adam8_R | AGGCCAAACCACTTCATACTG |
| mqRT_App_F | TCCGAGAGGTGTGCTCTGAA |
| mqRT_App_R | CCACATCCGCCGTAAAAGAATG |
| mqRT_Ar_F | TCCAAGACCTATCGAGGAGCG |
| mqRT_Ar_R | GTGGGCTTGAGGAGAACCAT |
| mqRT_cdh2_F | AGGCTTCTGGTGAAATTGCAT |
| mqRT_cdh2_R | GTCCACCTTGAAATCTGCTGG |
| mqRT_Egfr_F | GCCATCTGGGCCAAAGATACC |
| mqRT_Egfr_R | GTCTTCGCATGAATAGGCCAAT |
| mqRT_Map3k15_F | CCATGTGCTGACTATTTTTGCTG |
| mqRT_Map3k15_R | TCTTTCGCCAGTTCATCACCT |
| mqRT_Foxg1_F | AGCGACGACGTGTTCATCG |
| mqRT_Foxg1_R | CCCGTTGTAACTCAAAGTGCTG |
| mqRT_Nrk_F | GATAGGAAGACGGGTGAGAGT |
| mqRT_Nrk_R | GGCCAGGAGGATTCAGCTT |
| mqRT_Ntrk1_F | CAGTCTGATGACTTCGTTGATGC |
| mqRT_Ntrk1_R | CTCTTCACGATGGTTAGGCTTC |
| mqRT_Lefty1_F | CCAACCGCACTGCCCTTAT |
| mqRT_Lefty1_R | CGCGAAACGAACCAACTTGT |
| mqRT_Hoxd9_F | GCACCCTCAGCAACTACTACG |
| mqRT_Hoxd9_R | AAAACTACACGAGGCGAACTC |
| mqRT_Tbx15_F | GGCAATGCTGATTCCCCTGT |
| mqRT_Tbx15_R | CAAAACTGACCACCTGTCTCATC |
| mqRT_Wnt9a_F | GGCCCAAGCACACTACAAG |
| mqRT_Wnt9a_R | AGAAGAGATGGCGTAGAGGAAA |
| mqRT_Ccn2_F | GGCCTCTTCTGCGATTTCG |
| mqRT_Ccn2_R | GCAGCTTGACCCTTCTCGG |
